# Pharmacological and non-pharmacological interventions to influence adipose tissue function

**DOI:** 10.1186/1475-2840-10-13

**Published:** 2011-01-28

**Authors:** Jan Westerink, Frank LJ Visseren

**Affiliations:** 1 1Department of Vascular Medicine, University Medical Center Utrecht, the Netherlands

## Abstract

Obesity is associated with metabolic derangements such as insulin resistance, inflammation and hypercoagulobility which can all be understood as consequences of adipose tissue dysfunction. The potential role for adipose tissue derived cytokines and adipokines in the development of vascular disease and diabetes may produce a clinical need to influence adipose tissue function. Various pharmacological and non-pharmacological interventions affect plasma cytokine and adipokine levels. The effects of these interventions depend on weight loss *per se*, changes in fat distribution without weight loss and/or direct effects on adipose tissue inflammation.

Weight loss, as a result of diet, pharmacology and surgery, positively influences plasma adipokines and systemic inflammation. Several classes of drugs influence systemic inflammation directly through their anti-inflammatory actions. PPAR-γ agonism positively influences adipose tissue inflammation in several classes of intervention such as the thiazolidinediones and perhaps salicylates, CB1-antagonists and angiotensin II receptor blockers. Furthermore, within drug classes there are differential effects of individual pharmacologic agents on adipose tissue function.

It can be concluded that several commonly used pharmacological and non-pharmacological interventions have unintended influences on adipose tissue function. Improving adipose tissue function may contribute to reducing the risk of vascular diseases and the development of type 2 diabetes.

## Introduction

The prevalence of obesity is rising worldwide[1]. As obesity is a major cause of insulin resistance, metabolic syndrome, type 2 diabetes, atherosclerosis and premature death, the incidence of these diseases is expected to rise. Imbalance between caloric intake and energy expenditure leads to hypertrophy and hyperplasia of adipose tissue[2], leading to metabolic derangements, such as dyslipidemia, elevated blood pressure, inflammation, hypercoagulobility, as a consequence of adipose tissue dysfunction [3-5].

Adipose tissue dysfunction can be viewed as a combination of pro-inflammatory changes in adipose tissue and changes in the endocrine function of adipose tissue as witnessed by changes in plasma cytokine and especially plasma adipokine levels. Visceral adipose tissue (VAT) is the predominant adipose tissue compartment responsible for the production of proinflammatory cytokines and adipokines [6].

Various mechanisms are implicated in the initial phases of adipose tissue inflammation, most of which are a result of adipose tissue expansion[7]. The histopathology of adipose tissue inflammation in obesity is characterized by accumulation of macrophages in adipose tissue[8]. Subsequent adipose tissue inflammation leads to the systemic release of cytokines and adipokines by inflammatory cells, preadipocytes and adipocytes. Although most of the cytokines and adipokines are not solely produced by adipose tissue, they do reflect the vast production capacity of adipose tissue as exampled by the 30% contribution of adipose tissue on systemic IL-6 plasma levels[9]. As adipose tissue contributes significantly to systemic concentrations of cytokines and adipokines, plasma concentrations can therefore be regarded as a reflection of adipose tissue dysfunction.

The potential role for adipose tissue derived cytokines and adipokines in the development of vascular disease and diabetes[10-13] may produce a clinical need to influence adipose tissue function. Various pharmacological and non-pharmacological interventions, already commonly used in patients with vascular disease or type 2 diabetes affect adipose tissue function. The effects of these interventions may depend on weight loss but particularly on a loss of fat mass, changes in fat distribution without weight loss and/or direct effects on adipose tissue inflammation. In this paper we review the current pharmacological and non-pharmacological options and their possible underlying mechanisms for influencing adipose tissue function.

## Effects of weight loss on adipose tissue function

The quantity of adipose tissue is an important driver of adipose tissue dysfunction, insulin resistance and cardiovascular disease. Therefore, to improve adipose tissue function the logical and preferred first step should be to decrease the amount of (visceral) adipose tissue. Decreasing the amount of visceral adipose tissue may be accomplished via the following mechanisms: weight loss per se, loss of fat mass with an increase in fat-free mass (such as seen with exercise) or by inducing a shift in fat distribution from visceral to subcutaneous compartments. Although not a subject of this review, altering dietary content without altering the caloric content, such as by reducing fructose intake may also have beneficial effects on adipose tissue function [10]. For example, in an isocaloric diet, fructose rather than glucose intake was associated with an increase in visceral adipose tissue in humans [14] while fructose intake in mice induces visceral and perivascular adipose tissue dysfunction [15,16].

### Diet-induced weight loss

Intentional weight loss is associated with lowering blood pressure in hypertensive patients, improvements in the lipid profile and a decreased incidence of diabetes[17-19]. Intentional weight loss induced by taking dietary measures improves endothelial function[20-22], lowers systemic markers of inflammation[23] and ameliorates insulin resistance[24]. In patients with and without the metabolic syndrome, 7% weight loss already reduced the prevalence of components of the metabolic syndrome such as systolic blood pressure, plasma glucose levels, triglycerides and high-density lipoprotein (HDL) cholesterol[25]. Although there are no randomized controlled trials (RCT) available showing the benefit of diet induced weight reduction on overall or cardiovascular mortality there is a clear association between obesity and mortality[26-28]. The currently ongoing Look AHEAD trial compares intensive lifestyle intervention including weight loss with diabetes support and education and has shown promising results on diabetes control and cardiovascular risk factors[18]. Influencing plasma adipokines levels such as adiponectin by a dietary intervention seems especially dependent on a reduction in fat mass[29].

Lowering the daily caloric intake by 500 kilocalories leads to a decrease in bodyweight between 5-10% and a decrease in body mass index (BMI) by 2.0 and 2.9 kg/m2[30-32]. This weight loss was associated with a 25% fall in leptin plasma concentrations while there was no effect on adiponectin levels[30,32]. Weight loss of 5-10% increases insulin sensitivity while marginally increasing plasma levels of adiponectin and reducing plasma levels of C-reactive protein (CRP), interleukin-6 (IL-6) and the soluble tumour necrosis factor-α (TNF-α) receptor without an effect on plasma levels of TNF-α, indicating an improvement in adipose tissue function[31,32].

If caloric intake is further restricted by 600-1000 kcal/day, bodyweight decreases between 7 and 12%[25,33] resulting in a 46-58% fall in plasma leptin levels and an increase of 42-65% in adiponectin levels[25]. Apparently, diet-induced weight loss is an effective strategy for improving adipose tissue function but at least 10% weight loss is needed to improve plasma concentrations of adiponectin and inflammatory markers such as CRP.[34] Besides the amount of weight loss the duration of the weight loss period might also influence plasma adiponectin levels with adiponectin levels increasing during the weight loss maintenance period after a weight loss of 11-12% in 8 weeks[35].

### Medication-induced weight loss

#### Orlistat

Orlistat is a lipase inhibitor that decreases intestinal fat absorption after meals. Therefore, successful treatment with orlistat should be seen as a combined treatment of orlistat and a (low fat) diet. In a recent meta-analysis of 16 studies including 10,631 patients with a follow up of 1-4 years, orlistat reduced weight by 2.9 kg (95% CI 2.5-3.2 kg) and increased absolute percentages of participants achieving 5% and 10% weight loss thresholds by 21% and 12% respectively[36]. A decrease in the incidence of type 2 diabetes mellitus from 9.0% to 6.2% (Hazard Ratio 0.63; 95% CI 0.46-0.86) was reported.[37] Together with a dietary intervention, orlistat (120 mg tid.) was not associated with a change in plasma leptin and adiponectin concentrations, although resistin levels decreased by 36% after 6 months of treatment.[38] When orlistat was combined with a hypocaloric diet with a 600 kcal restriction, bodyweight decreased by 14-24%, percentage body fat by 21% and plasma concentrations of leptin, CRP, IL-6, TNF-α, and resistin decreased while adiponectin increased, indicating an improvement in adipose tissue function[39,40].

#### Sibutramine

Sibutramine is a highly selective inhibitor for the reuptake of norepinefrine and serotonin at nerve endings. Originally developed as an anti-depressant, sibutramine has effects on energy intake and to a lesser extent on energy expenditure. The latter is probably mediated by brown adipose tissue thermogenesis[41]. In a meta-analysis of placebo-controlled randomized trials, sibutramine decreased bodyweight by 4.2 kg (95% CI 3.6-4.7 kg)[36]. Compared with placebo, sibutramine however increased systolic blood pressure by 1.7 mmHg (95% CI 0.1-3.3) and pulse rate by 4.5 beats/min (95% CI 3.5-5.6). Other common side effects included dry mouth, insomnia and nausea in 7-20%.

Two small studies evaluated the combined effect of a 500-600 kcal diet restriction and sibutramine (10-15 mg daily). Bodyweight fell by only 5-7%, but plasma concentrations of TNF-α, IL-6, resistin, leptin and CRP levels decreased[38,42]. In contrast to diet only, the combination of a diet with sibutramine was associated with an increase in adiponectin and interleukin-10 (IL-10) levels while only inducing a relatively small weight loss[42]. Also, a 7% fall in bodyweight and 14% fall in fat mass due to sibutramine was associated with improved insulin resistance and a rise in adiponectin which was especially correlated with the decrease in visceral adipose tissue area [43]. The effect of sibutramine induced weight loss on adipokines seems greater than in studies in which caloric restriction is used. The question therefore arises whether sibutramine may have effects on adipose tissue function independent of sole weight loss. Catecholamine induced lipolysis has been shown to be greater in visceral adipose tissue (VAT) then in subcutaneous adipose tissue (SAT)[44]. Indeed, combining a diet with sibutramine led to a preferential loss of VAT in patients with obstructive sleep apnea[45]. This potential preferential effect on VAT may be an explanation for the greater effect of sibutramine on adipokines and cytokines than could be expected by similar weight loss with a diet alone. However, recently it has been shown that use of sibutramine is associated with an increased risk for non-fatal myocardial infarction and stroke in patients at high cardiovascular risk[46]. Since October 2010 sibutramine has therefore been withdrawn from the market.

#### Cannabinoid-1 (CB1) receptor antagonists

The cannabinoid-1 (CB1) receptor is widely dispersed throughout the body with high concentration in areas of the brain related to feeding[47]. CB1 receptors are also present on adipocytes[48]. The two best characterized endocannabinoids, anandamide (AEA) and 2-arachnidonylglycerol (2-AG) are both capable of activating the peroxisome proliferator-activated receptor (PPAR) α and γ[49]. This activation may be induced by direct binding to PPAR or by intracellular signalling after CB receptor activation (mediated by extracellular signal-regulated kinase 1/2 (ERK1/2) and p38 mitogen-acivated protein kinase (MAPK)) or by COX-2 metabolites of endocannabinoids[49,50].

Interestingly enough, peripheral endocannabinoid levels are increased in human obesity, probably due to diminished insulin mediated down regulation of the endocannabinoid system in insulin resistant adipocytes[51,52]. The selective CB1 receptor blocker rimonabant has been investigated as a weight reduction drug in several large scale clinical trials [53-56]. Rimonabant not only has central effects on satiety but also affects the peripheral endocannabinoid system in the gut leading to nausea and diarrhoea, all of which can explain the rimonabant associated weight loss[57]. In a recent meta-analysis of placebo-controlled trials, evaluating the clinical effects of rimonabant, it appeared that the mean weight loss was 4.7 kg (95% CI 4.1-5.3) more than in the placebo group[36]. Furthermore, rimonabant significantly reduced waist circumference, lowered blood pressure, lowered triglyceride levels and increased high density lipoprotein cholesterol plasma concentrations. Although rimonabant was withdrawn from the market in 2008 due to adverse effects including an increased incidence of psychiatric disorders (depression, suicidal ideation, anxiety, and aggression), other CB1 receptor antagonists are still under investigation.

In patients with type 2 diabetes, treatment with rimonabant at the highest dose (20 mg) decreased CRP (-26%) and leptin (-2%)levels[55]. In overweight or obese patients with untreated dyslipidemia, rimonabant decreased leptin levels to a greater extent than in patients with diabetes (23%) and significantly increased adiponectin levels by 37%[53]. In regression analysis 57% of the 1-year treatment effect of rimonabant on adiponectin was thought not to be attributable to weight loss [58]. Although not completely explained, these data suggest that rimonabant may have effects on adiponectin levels beyond weight loss. Adipocytes express a CB1 receptor and may therefore be a direct target for rimonabant[48]. Also, unbound circulating endocannabinoids may still activate PPARs and may therefore provide an explanation for weight loss independent effects of rimonabant on adipose tissue function.

#### Bariatric surgery

Bariatric surgery is increasingly used as a strategy to reduce bodyweight and thereby ameliorate risk factors for cardiovascular disease[59-61]. On average, patients lose 14-25% weight after bariatric surgery[62]. Patients who underwent gastric bypass surgery showed a significant decline in all-cause mortality as well as coronary artery disease, diabetes and cancer during 7.1 years follow up[26]. Patients with recently diagnosed type 2 diabetes showed greater weight loss after gastric banding compared to conventional therapy (life style advice) as well as a greater chance of remission of type 2 diabetes[63]. This effect of bariatric surgery on diabetes is probably due to a reduction in body fat mass and, in the case of gastric bypass surgery, changes in gut hormone production such as Glucagon-Like Peptide-1 (GLP-1), Gastric Inhibitory Polypeptide (GIP) and grehlin[64]. GLP-1 receptor agonists induce adiponectin expression while reducing expression of IL-6 and MCP-1 in 3T3-L1 adipocytes through the protein kinase A pathway[65]. Finally, although only limited data is present, plasma GIP levels may important as seen from in vitro studies showing a GIP induced reduction in insulin resistance in 3T3-L1 adipocytes through activation of Akt[66]. Although only limited data is available, the effects of GLP-1 and GIP on adipocytes may be part of the weight loss independent effects of gastric bypass surgery on adipose tissue function. Other beneficial effects of gastric bypass surgery in comparison to gastric banding may include a smaller fat mass to fat-free mass ratio with similar weight loss[67].

Adiponectin levels have been shown to increase after bariatric surgery in several small scale studies mainly because of an increase in high molecular weight adiponectin[68-70]. After bariatric surgery, plasma concentrations of Macrophage Inhibitory Factor (MIF), Plasminogen Activator Inhibitor-1 (PAI-1), Retinol Binding Protein-4 (RBP-4), Monocyte Chemotactic Protein-1 (MCP-1) and interleukin-18 (IL-18) are decreased, indicating positive effects on adipose tissue function[71-73].

## Effects of exercise on adipose tissue function

The most important reason why insulin resistance increases with age is the steady increase in bodyweight and the reduction in physical activity[74]. Comparison of studies investigating the effect of exercise and diet on body weight is hampered by the different exercise schedules, combination with different dietary restrictions and different patient groups studied. Exercise does not lead to preferential loss of VAT when comparing moderate and vigorous exercise with dietary caloric restriction, nor is there a clear difference in the change of body composition [75] Both aerobic and strength exercise lead to an amelioration of insulin resistance[24]. Even short duration exercise improves insulin resistance, suggesting that some beneficial effects of exercise are not mediated by weight loss[76].

In patients with type 2 diabetes mellitus, aerobic exercise during 16 weeks was associated with a decrease in bodyweight by only 1.3 kilograms and by lower plasma concentrations of IL-6, IL-18, CRP and resistin showing an anti-inflammatory effect of exercise with only marginal weight reduction[77]. Exercise may improve adipose tissue function in healthy patients with the largest effect in the older age group as shown by an increase in plasma adiponectin levels and a decrease in plasma RBP-4 levels[78]. A recent systemic review however showed that most but not all studies investigating the effect of exercise failed to show an effect on circulating adiponectin levels probably due to the only limited weight loss associated with pure exercise studies[78,79]. These findings are compatible with data from studies investigating diet induced weight loss which showed that more than 10% weight loss is needed to elevate plasma adiponectin levels[34].

## Effects of pharmacologic agents on adipose tissue function

### Salicylates

Salicylates are among the most commonly used nonsteroidal anti-inflammatory drugs and have their main action through cyclooxygenase (COX) inhibition. Studies investigating the effects of salicylates on adipose tissue function have especially implicated specific COX-2 inhibition as the mechanism by which salicylates may improve adipose tissue function[80,81]. Besides COX inhibition, salicylates also act through inhibition of the activity of inhibitor of nuclear factor kappa-B kinase subunit beta (IKK-β) leading to decreased phosphorylation of inhibitor of NF-κB (IκB) and therefore to a reduction in translocation of Nuclear Transcription factor kappa-B (NF-κB) to the nucleus [82]. Besides the direct anti-inflammatory effects, some data suggests a possible role of PPAR-γ agonism which is of great importance in adipocyte differentiation, function and body fat composition. For example, 5-aminosalicylic acid increases PPAR-gamma expression, promotes its translocation from the cytoplasm to the nucleus, and permits the recruitment of co-activators and the activation of a peroxisome-proliferator response element-driven gene in human epithelial cells[83]

Although high dose acetylsalicylic acid (1 to 1,6 gram) has been shown to reduce fasting and post load glucose levels in patients with type 2 diabetes, the clinical use of high dose acetylsalicylic acid is limited by the increased risk of bleeding)[84]. Low dose acetylsalicylic acid (100 mg and 300 mg) had no effect on IL-6 or CRP levels in patients with type 2 diabetes during 6 weeks[85]. Salsalate at a dose of 3 grams per day however does lower fasting glucose levels and glucose levels after a oral glucose tolerance test in patients with obesity, by increasing insulin levels via an unknown mechanism[86]. In patients with type 2 diabetes, salsalate in doses of 3 and 4,5 grams per day improved insulin resistance as measured during a hyperinsulinemic euglycemic clamp, fasting and post challenge glucose levels, decreased free fatty acid (FFA) levels and increased adiponectin levels by 35-45% without an effect on bodyweight[87]. This effect of salsalate on adipose tissue dysfunction may be mediated by the before mentioned anti-inflammatory effect or by the possible PPAR-γ agonistic action of salicylates, leading to a reduction in insulin resistance.

### Beta-blockers

Although earlier reports have found a possible link between the use of beta blockers and the development of diabetes, some newer beta-blockers are investigated for their beneficial effects on adipose tissue dysfunction[88,89]. The relation between beta-blockers and diabetes can be explained by β_2 _receptor blockade, induced reduction in thermogenesis and subsequent weight gain [90-92]. A combined β1 and β2-adrenoceptor agonist is capable of down regulating adiponectin and up regulating TNF-α mRNA in murine adipocytes[93].

Indeed, some of the newer beta blockers do have beneficial effects on insulin resistance and adipokines without changes in weight. For example nebivolol (5 mg daily), which has β_2 _intrinsic sympaticomimetic action, increases plasma adiponectin levels in overweight patients with hypertension[94] Celiprolol (up to 400 mg daily), a combined β_1 _antagonist and β_2 _agonist reduces plasma leptin levels without a change in bodyweight in patients with dyslipidemia [95].

### Aldosterone antagonists

Inhibition of mineralcorticosteroid receptor activation by use of aldosterone antagonists is used in the treatment of heart failure and hypertension. Besides an effect on blood pressure, spironolactone is capable of inhibiting TNF-α, IL-6 and Interferon- γ (IFN-γ) production in isolated human mononuclear cells in vitro[96]. Far less is known about the effect of aldosterone antagonists on adipose tissue dysfunction. Adipose tissue is capable of producing an unidentified mineralcorticoid releasing factor that may stimulate aldosterone production[97]. The mineralcorticoid receptor has an important role in adipocyte differentiation as witnessed by diminished differentiation of 3T3-L1 adipocytes in the presence of dexamethason and spironolactone [98]. In obese diabetic mice, blocking the mineralcorticoid receptor reduced the expression of proinflammatory cytokines in adipose tissue while it lead to an increased expression of adiponectin in cardiac and adipose tissue[99]. Further evidence for an important role for the mineralcorticoid receptor in adipose tissue comes from a study in obese mice where blocking the mineralcorticoid receptor with eplererone ameliorated insulin resistance, decreased the number of hypertrophic adipocytes and infiltrating macrophages[100]. Furthermore, eplererone was also capable of blunting aldosterone and H_2_O_2 _induced radical oxygen species and dysregulated expression of obesity related genes in 3T3-L1 adipocytes. This data from in vitro and murine studies shows that aldosterone might play a relevant role in adipocyte biology. Indeed, although not a strict adipokine, plasma PAI-1 levels were reduced by spironolactone in patients with type 2 diabetes and diabetic nephropathy[101].

### Angiotensin Converting Enzyme Inhibitors (ACE-i)

Angiotensin Converting Enzyme Inhibitors (ACE-i) are widely used in the treatment of heart failure and hypertension. In a large randomized trial, ramipril was associated with a lower incidence of diabetes, compared to placebo, in patients at high cardiovascular risk [102]. In patients with cardiovascular disease and impaired fasting glucose, ramipril did not reduce the incidence of diabetes mellitus but was associated with regression to normoglycaemia[103].

Circulating levels of angiotensin II are associated with changes in VAT in humans[104]. ACE-inhibitors may affect insulin resistance by reducing plasma concentrations of angiotensin II. Angiotensin II increases serine phosphorylation of the insulin receptor, insulin receptor substrate 1 and phophadidylinositol-3-kinase leading to a state of insulin resistance[105]. Angiotensin II might also influence insulin resistance via a direct pro-inflammatory effect on adipocytes and subsequent changes in MCP-1, IL-6 and IL-8 production via the NF-κB pathway and increased production of leptin via an ERK1/2 dependent pathway in a murine model[106-108]. Finally, ACE-i decreases total body fat mass and plasma leptin levels in a murine model[109].

Lisinopril binds to PPAR-γ, although with a low binding affinity, suggesting a possible role for a PPAR-γ agonistic action for ACE-i[110]. ACE-i are less effective than angiotensin II type 1 receptor blockers (ARB) in raising plasma adiponectin levels[111-113], which is likely a consequence of the different effects on PPAR-γ.

### Angiotensin II type 1 Receptor Blockers (ARB)

The ARB valsartan reduces the risk of developing type 2 diabetes mellitus in patients with hypertension[114]. In clinical studies it is shown that insulin resistance is indeed reduced by the use of ARBs[115,116]. Apart from blockade of the angiotensin II type 1 receptor, ARBs function as partial agonists of PPAR-γ, even in the absence of a functional AT-II receptor[117,118]. In a murine model, plasma adiponectin levels were elevated after treatment with irbesartan without a change in adiponectin mRNA levels, suggesting a post-transcriptional mechanism[119]. The effect on PPAR-γ is further shown by studies investigating the effect of ARBs on adipose tissue distribution. Telmisartan decreases VAT by 10%, as measured by CT, without having an effect on subcutaneous fat area[120,121]. ARBs also have anti-inflammatory effects as seen by lowering effect on plasma TNF-α and IL-6 levels in patients with diabetes and hypertension[120,122] Telmisartan, but not valsartan, was shown to attenuate TNF-α induced IL-6 production by vascular smooth muscle cells in a PPAR-γ dependent manner[123]. These PPAR-γ agonistic effects of ARBs result in higher plasma levels of adiponectin[120,121,124], although no effect was observed on high-molecular weight adiponectin levels[115,116].

### Statins

Statins might have a various direct effects on adipose tissue function by inhibiting Toll-like receptor-4 (TLR4) triggered expression of IFN-γ in macrophages, which are abundant in adipose tissue, and through increasing PPAR- γ expression[125,126]. Besides direct effects on adipose tissue, statins are also capable of reducing inflammation in general as measured by reduced plasma CRP levels[127]. Incubation of murine 3T3-L1 adipocytes with blood samples from patients treated with pravastatin induced adiponectin production[128]. However, pravastatin, a hydrophilic statin[129], did not alter insulin sensitivity, or leptin and adiponectin plasma concentrations in healthy subjects[130]. Pravastatin treatment however did elevate plasma adiponectin levels more in patients with lower baseline levels compared to patients with higher baseline adiponectin plasma concentrations[131,132].

Due to differences in lipophylicity, statins may have different effects on adipose tissue function. Atorvastatin, which is more lipophylic than pravastatin[129] increases adiponectin levels in patients with coronary artery disease (CAD) or at high risk for CAD while having no effect on adiponectin in patients with diabetes [133-136]. Simvastatin, the most lipophylic statin, decreases adiponectin [137,138]. Rosuvastatin, a very hydrophilic statin, was able to lower visfatin levels in patients with increased risk for cardiovascular disease while simvastatin had no effect[139,140]. This suggests a beneficial effect of hydrophilic statins over lipophylic statins on adipose tissue dysfunction. Initially statins were thought to reduce the incidence of diabetes[141], but two recent meta analyses of statin trials on the incidence of diabetes showed that there is no or even a small increased risk of diabetes due to statin treatment without clear heterogeneity between statins[142,143].

### Fibrates

Fibrates decrease the incidence of type 2 diabetes mellitus[144], by a PPAR-α agonistic effect[145]. The PPAR-α agonistic effects of fibrates also include an anti-inflammatory regulatory action on macrophages by interfering with the NF-κB and AP-1 pathways[146]. Besides the PPAR-α agonistic effect, some fibrates like bezafibrate, can be seen as pan-PPAR agonists and therefore may have effects through PPAR-γ and/or PPAR-β/δ[147,148] As mentioned before, this could be significant as especially PPAR-γ is of major importance for adipocyte differentiation and function. Fibrates inhibit the expression of PAI-1 in human adipocytes and preadipocytes, an effect which is blunted when cells are co-incubated with a PPAR-α inhibitor[149]. In a murine model, fenofibrate increased adiponectin and visfatin mRNA levels while decreasing the expression of TNF-α in the VAT without an effect on serum TNF-α levels[150]. Short-term treatment effects of fibrates on adipose tissue function are seen by lower TNF-α, IL-6, PAI-1, MCP-1 and RBP-4 plasma levels during treatment[151,152], and by an increase in high molecular weight adiponectin levels by 12% in patients with hypertriglyceridemia[153].

### Thiazolidinediones

Peroxisome Proliferator-Activated Receptors or PPARs are ligand-activated transcription factors that belong to the nuclear receptor superfamiliy. While the thiazolidinedione (TZD) rosiglitazone is a selective PPAR-γ agonist, pioglitazone exerts PPAR-γ and -α agonistic activity which may account for the differential metabolic effects of pioglitazone and rosiglitazone. Thiazolidinediones have been investigated as potential drugs in preventing type 2 diabetes. Treatment with rosiglitazone during 3 years lowered the incidence of diabetes mellitus type 2 (HR 0.38, 95% CI 0.33-0.44).[154].

Thiazolidinediones may directly increase insulin sensitivity in the liver and adipose tissue where it is of critical importance for adipocyte differentiation. Indeed, as a consequence of PPAR-γ agonism, thiazolidinediones increase SAT-mass[155]. PPAR-γ agonists are thought to promote free fatty acid uptake and storage in adipocytes and may therefore protect the liver and muscle from excess levels of free fatty acids and their toxic effects, resulting in insulin resistance. Also, PPAR-γ agonists may have indirect effects on insulin resistance by altering adipocytokine production. Pioglitazone increases high molecular weight adiponectin and decreases TNF-α levels and RBP-4 levels in patients with type 2 diabetes[155-157].In addition, this effect of pioglitazone on plasma levels of adiponectin is highly predictable on baseline levels[158]. Rosiglitazone increases leptin levels as would be expected due to the expansion of the SAT compartment and has effects on adipocytokine production as shown by lowering PAI-1 levels, which is partly dependent on adiponectin, and increasing adiponectin plasma levels [159-161]. Although PPAR-γ agonists have shown considerable beneficial effects on adipose tissue function, concerns about cardiovascular safety remain. Both thiazolidinediones are associated with a 3-4 kg increase in bodyweight probably due to fluid retention which leads to an increased risk for heart failure[162,163] Rosiglitazone therapy is associated with an increased risk for the occurrence of myocardial infarctions which has lead to the withdrawal of this drug from the market in 2010[164]. As beneficial vascular effects are seen with pioglitazone, current research is focussing on other dual PPAR-α/γ agonists to improve not only glycemic control but also lipid levels and potentially reducing vascular risk[165].

### Metformin

Metformin reduces the incidence of type 2 diabetes in patients with elevated fasting and post-load glucose concentrations indicating an effect of metformin in reducing insulin resistance[166]. Apart from affecting glucose uptake in the liver and in peripheral tissues, metformin has anti-inflammatory properties by inhibiting NF-κB and blocking the PI3K-Akt pathway in human vascular wall cells[167]. Recent evidence suggests a possible role of metformin on AMP-activated protein kinase dependent lipolysis in adipocytes which may lead to lower plasma levels of fatty acids and therefore to improvement in adipose tissue function[168]. Production of PAI-1 by human subcutaneous adipose tissue (SAT) is inhibited by metformin in vitro, showing a potential direct effect of metformin on adipose tissue function[169]. However in a study with lean and obese patients with and without diabetes, metformin did not result in a reduction of BMI, nor did it affect plasma adiponectin levels after 4 months of treatment[170] Other studies have shown that metformin decreases plasma concentrations of MIF in obese patients and also decreases vaspin while increasing omentin plasma concentrations in overweight women with polycystic ovary syndrome, without an effect on bodyweight[171-173]. These results indicate a direct effect of metformin on adipose tissue in humans, beyond an effect through weight reduction. Apart from these direct effects on adipose tissue function, metformin may also work through effects on body composition. Metformin does not affect the amount of VAT, but reduces SAT, total body fat percentage, BMI and waist circumference in obese children and adolescents[174]. It can be concluded that metformin, which has an important place in the treatment of type 2 diabetes, also has direct beneficial effects on adipose tissue function.

## Conclusion

Obesity related adipose tissue dysfunction may be an important risk factor for the development of vascular diseases and diabetes. Weight reduction and physical exercise improve adipose tissue function, probably to a large extent due to a reduction in fat mass. Various pharmacological agents commonly used in patients with vascular diseases or diabetes mellitus also affect adipose tissue function by various mechanisms (Table 1). Within classes of drugs there are differential effects of individual pharmacological agents. Although the effects of these drugs on adipose tissue function are unintended, improving adipose tissue function may contribute to reducing the risk of vascular diseases and the development of type 2 diabetes. However, the clinical relevance of influencing adipose tissue function remains to be determined.

**Table 1:** Differential effects of currently available interventions in the treatment of adipose tissue dysfunction

**Intervention**	**Mechanism of action responsible for the effect on cytokines and adipokines**	**Effect on cytokines and adipokines**
Diet	Weight loss	Adiponectin↑, leptin↓, IL-6↓, sTNF-α receptor type 1↓
Orlistat	Weight loss	Adiponectin↑, resistin↓, leptin↓, IL-6↓, TNF-α↓
Sibutramine	Weight loss	Adiponectin↑, resistin↓, leptin↓, IL-10↑, TNF-α↓, IL-6↓
CB1-antagonists	Weight loss	Adiponectin↑, leptin↓
	Effects through PPARγ activation	
Bariatric surgery	Weight loss	Adiponectin↑, HMW adiponectin↑, RBP-4↓, PAI-1↓, MIF↓, MCP-1↓, IL-18↓
	Effects on gut hormones	
Exercise	Weight loss	Adiponectin↑, resistin↓, RBP-4↓, IL-6↓, IL-18↓
Salicylates	Anti-inflammatory	Adiponectin↑
	COX-2 inhibition	
β-blocker	β_2 _adrenoreceptor activation	Adiponectin ↑, leptin↓
Aldosteron antagonists	Aldosteron antagonism	PAI-1↓
Angiotensin Converting Enzyme inhibitors	Inhibition of the renin-angiotensin system	Adiponectin↑
Angiotensin II type 1 receptor blockers	Inhibition of the renin-angiotensin system	Adiponectin↑, TNF-α↓, IL-6↓
	Effects through PPARγ activation	
(HMG-CoA) reductase inhibitors	Anti-inflammatory	Adiponectin↑↓, visfatin↓
Fibrates	Effects through PPARα activation	Adiponectin↑, RBP-4↓, PAI-1↓, MCP-1↓, TNF-α↓, IL-6↓
	Effects through PPARγ activation	
Metformin	Anti-inflammatory	Vaspin↓, MIF↓
	AMPK activation	
Thiazolidinediones	Effects through PPARγ activation	Adiponectin↑, HMW adiponectin↑, leptin↑, RBP-4↓, PAI-1↓

AMPK: AMP activated protein kinase, CB-1 antagonists: Cannabinoid-1 antagonist, IL: Interleukin, MCP-1: Monocyte chemoattractant protein-1, MIF: Macrophage Inhibitory Factor, PAI-1: Plasminogen activator inhibitor-1, PPAR: Peroxisome Proliferator Activated Receptors, RBP-4: Retinol Binding Protein 4, TNF-α: Tumor Necrosis Factor-α

## Abbreviations

2-AG: 2-Arachnidonyl Glycerol; ACE: Angiotensin Converting Enzyme; AEA: Anandamide; ARB: Angiotensin II type 1 receptor blocker; AT-II: Angiotensin II; BMI: Body Mass Index; CAD: Coronary Artery Disease; CB-1: Cannabinoid-1; COX: Cyclooxygenase; CRP: C-Reactive Protein; ERK1/2: Extracellular signal-Regulated Kinas 1/2; FFA: Free Fatty Acids; GIP: Gastric Inhibitory Polypeptide; GLP-1:Glucagon-Like Peptide 1; HDL; High Density Lipoprotein; IFN-γ: Interferon-γ; IL: Interleukin; MAPK: Mitogen-Activated Protein Kinase; MCP-1: Monocyte Chemoattractant Protein 1; MIF: Macrophage Inhibitory Factor; PAI-1: Plasminogen Activator Inhibitor 1; PPAR: Peroxisome Proliferator-Activated Receptor; RBP-4: Retinol Binding Protein 4; SAT: Subcutaneous Adipose Tissue; TLR-4: Toll-Like Receptor 4; TNF-α: Tumour Necrosis Factor-α; TZD: Thiazolidinediones; VAT: Visceral Adipose Tissue;

## Competing interests

The authors declare that they have no competing interests.

## Authors' contributions

JW has designed the review, acquired the data and drafted the manuscript

FLJV has been involved in revising the article critically for important intellectual content and gave final approval of the version to be published.

Both authors have read and approved the final manuscript.

## Acknowledgements

This work was supported by a grant from the Leatare Foundation, Monaco and the Catharijne Foundation, the Netherlands.
